# Encouraging impact following 2.5 years of reinforced malaria control interventions in a hyperendemic region of the Republic of Guinea

**DOI:** 10.1186/s12936-016-1353-z

**Published:** 2016-05-28

**Authors:** Amanda Tiffany, Faya Pascal Moundekeno, Alexis Traoré, Melat Haile, Esther Sterk, Timothée Guilavogui, Blaise Genton, Micaela Serafini, Rebecca F. Grais

**Affiliations:** Epicentre, Geneva, Switzerland; Médecins Sans Frontières, Guéckédou, Guinea; District Préfectoral de la Santé, Guéckédou, Guinea; Médecins Sans Frontières, Conakry, Guinea; Médecins Sans Frontières, Geneva, Switzerland; National Malaria Control Programme, Conakry, Guinea; Swiss Tropical and Public Health Institute, Basel, Switzerland; Infectious Disease Service and Department of Ambulatory Care, University Hospital Lausanne, Lausanne, Switzerland; Epicentre, Paris, France

**Keywords:** *Plasmodium falciparum*, Malaria, Prevalence, Symptomatic, Guinea, Hyperendemic, Cross sectional surveys

## Abstract

**Background:**

Malaria is one of the principal causes of morbidity and mortality in the Republic of Guinea, particularly in the highly endemic regions. To assist in malaria control efforts, a multi-component malaria control intervention was implemented in the hyperendemic region of Guéckédou Prefecture. The coverage of the intervention and its impact on malaria parasite prevalence were assessed.

**Methods:**

Five cross-sectional surveys using cluster-based sampling and stratified by area were conducted from 2011 to 2013 in three sous-préfectures of Guéckédou Préfecture that received the intervention: Guéckédou City, Tékoulo and Guendembou in addition to one comparison sous-préfecture that did not receive the intervention, Koundou. Surveys were repeated every 6 months, corresponding with the dry and rainy seasons. Rapid diagnostic tests (RDT) were used to diagnose malaria infection. In each selected household, bed net use and ownership were assessed.

**Results:**

A total of 35,123 individuals participated in the surveys. Malaria parasite prevalence declined in all intervention sous-préfectures from 2011 to 2013 (56.4–45.9 % in Guéckédou City, 64.9–54.1 % in Tékoulo and 69.4–56.9 % in Guendembou) while increasing in the comparison sous-préfecture (64.5–69 %). It was consistently higher in children 5–14 years of age followed by those 1–59 months and ≥15 years. Indicators of intervention coverage, the proportion of households reporting ownership of at least one bed net and the proportion of survey participants with fever who received treatment from a health facility or community health worker also increased significantly in the intervention areas.

**Conclusions:**

Implementation of the multi-component malaria control intervention significantly reduced the prevalence of malaria in the sous-préfectures of intervention while also increasing the coverage of bed nets. However, malaria prevalence remains unacceptably high and disproportionately affects children <15 years of age. In such situations additional vector control interventions and age specific interventions should be considered.

## Background

Malaria is endemic with perennial transmission in the Republic of Guinea (Guinea) where it is among the primary causes of morbidity and mortality for the population, responsible for 34 % of all medical consultations in 2012 [1]. Malaria prevalence is estimated to be 44 % nationally, although there are important regional differences in endemicity, with transmission highest in the heavily forested southern part of the country [1]. In Guinea the National Malaria Control Programme recommends the use of artemisinin combination therapy (ACT) for treatment for uncomplicated malaria. [2]. Malaria rapid diagnostic tests (RDT) and ACT are free of charge for the population in health facilities while microscopy services and other medications incur a fee.

Historically, epidemiological data are either inaccurate or sparse in Guinea, posing a challenge to use of health facility data for disease surveillance and monitoring programme impact. Prior to receiving support to surveillance from external partners and improved data collection from 2014 onwards, reporting of malaria cases was weak and consequently the malaria burden may have been severely underestimated in the country. In 2012, only 211,157 cases and 108 deaths due to malaria were reported in a population of 11.75 million [3]. This lack of reliable data hinders prevention and treatment efforts and requires improved data collection over longer periods in order to document trends and better understand the malaria burden in Guinea and, by extension, similar settings.

Impact from malaria control programmes results from the additive effects of multiple interventions and, when implemented with high coverage, they are expected to have greater impact than any one intervention alone [4]. Many controlled trials have been carried out to assess the efficacy of different malaria control interventions, including insecticide-treated nets [5], indoor residual spraying [6] and malaria intermittent preventive treatment in pregnancy [7, 8]. While curative interventions such as community case management of malaria have been documented to be effective [9] few have been tested in controlled trials. Additionally, little research has been carried out to investigate the additive or potentially synergistic effects of several interventions when implemented together under trial conditions [10, 11] or outside of trial conditions.

In collaboration with the Ministry of Health and National Malaria Control Programme, Médecins Sans Frontières (MSF) reinforced malaria control activities in Guéckédou Préfecture beginning in 2011. Guéckédou Préfecture was chosen to benefit from the reinforced activities based on the populations’ relatively poor access to health care and the (suspected) disproportionately high malaria burden in the region. All sous-préfectures within Guéckédou Préfecture were to be covered by the intervention package with implementation occurring in a stepwise manner. However, due to operational constraints implementation was ultimately restricted to the three sous-préfectures that received the intervention during the first phase of project rollout. In 2014 all activities were to be handed over to the Ministry of Health for continuation and expansion however the programme ended earlier than anticipated due to an outbreak of Ebola virus disease in the region.

The data presented here represent the first published description of the burden of malaria in Guéckédou Préfecture and evaluation of the impact of a malaria control intervention package on malaria burden in the same area.

## Methods

### Nature of the intervention

**Fig. 1:**
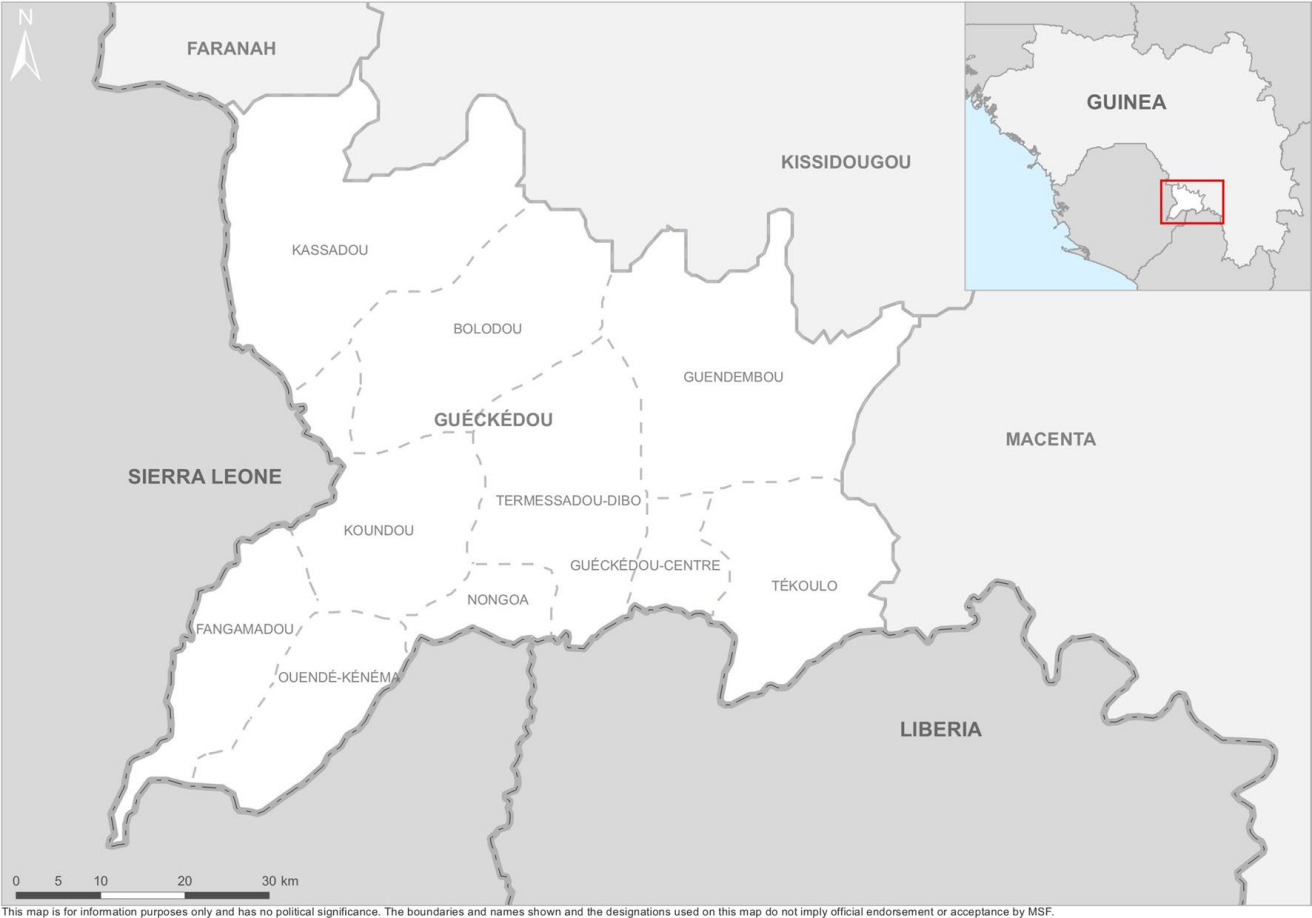
Map of Guéckédou prefecture

Administratively, Guinea is subdivided into eight regions comprised of 33 prefectures. Each préfecture is further divided into sous-préfectures [2]. The malaria intervention was intended to cover 3 of the 12 sous-préfectures in Guéckédou Prefecture, a geographic area of 1779 km^2^ with difficult access, particularly during the long rainy season when many roads to outlying villages become impassable. Activities were carried out from 2011 to 2014 in the intervention Sous-préfectures of Guéckédou City (urban), Tékoulo (rural) and Guendembou (rural) as seen in Fig. 1. The total population in these areas in 2010 was estimated at 224,399 individuals (estimates by the Sous-préfecture).

In line with strategy recommendations from the Roll Back Malaria partnership, the malaria control activities (intervention package) implemented in Guéckédou Préfecture involved both curative and preventive components [12]. Curative components included improving detection of clinical malaria cases and timely treatment with an artemisinin-based combination. This was done by ensuring malaria rapid diagnostic tests (RDT) and ACT were available and free of charge in all 21 MSF supported public health facilities in the intervention Sous-préfectures. Additionally, community health workers (CHW) were trained to use and interpret RDTs, treat RDT positive patients with an ACT and pre-treat and refer cases of severe malaria. Injectable artesunate was also introduced in the hospital as treatment for cases of severe malaria. Preventive activities included community health promotion designed to reinforce messages regarding the importance of being tested and treated for malaria. Long-lasting insecticide-treated nets (LLINS) were distributed in April 2012 during a mass campaign. LLINs were also provided to pregnant women during antenatal visits throughout the intervention period. MSF also ensured supply of sulfadoxine-pyrimethamine (SP) for malaria intermittent preventive treatment in pregnancy (IPTp) in all health facilities through September 2013. From October 2013, IPTp was replaced by intermittent screening and treatment for pregnant women in urban facilities while rural facilities continued with IPTp.

### Study design and target population

**Fig. 2:**
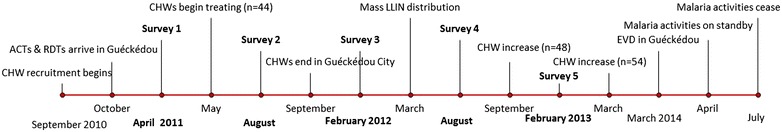
Timeline of intervention implementation and cross-sectional surveys 2010–2014

Five cross-sectional surveys were conducted from April 2011 to February 2013 to evaluate the impact of the malaria intervention package. Surveys were carried out during the peak of the rainy season (July/August) and dry season (February/April) in order to document any seasonal heterogeneity in malaria parasite prevalence. In addition to the three intervention sous-préfectures, a fourth sous-préfecture that did not receive the intervention package was selected to serve as a comparison sous-préfecture. The first survey was carried out in April 2011 in all four sous-préfectures while preparation for implementation of the intervention package was underway. The first survey was followed by four surveys in the same sous-préfectures in August 2011, February 2012, August 2012 and February 2013 (Fig. 2).

Households were selected using two-stage cluster sampling, stratified by Sous-préfecture. Thirty clusters, villages (rural) or neighbourhoods (urban), were randomly selected in each stratum with probability proportional to population size. Households were selected using the EPI method [13] within each cluster. Cluster selection carried out independently for each survey round. All household residents >1 month of age were included in the survey until at least 55 individuals were surveyed per cluster. Rapid diagnostic tests based on detection of the histidine rich protein-2 (Malaria Antigen P.f. SD Bioline^®^) were performed for all participants.

Sample size was estimated based on a hypothesized RDT malaria parasite prevalence of at least 50 % in each Sous-préfecture. Detecting a change of at least 10 % in malaria prevalence after 1 year of intervention with alpha of 5 %, 80 % power and a design effect of two required that 814 individuals be surveyed per sous-préfecture (strata). A stratified analysis by age required two times this number for a total sample size of 1650 individuals per strata or 6600 individuals in total, requiring a minimum of 55 individuals to be surveyed per cluster.

### Training and data collection

Each survey was conducted over 21 days by seven teams, each composed of one nurse and one laboratory technician who were accompanied by a driver. All team members underwent 5 days of training prior to the survey: 3 days of role-specific training followed by 1 day of joint team training and 1 day to pilot and practice the survey questionnaire, procedures and laboratory materials.

Data were gathered through face-to-face interviews for participants ≥15 years of age, primary caregivers were interviewed on behalf of children <15 years of age. Selected households that were found to be empty (but not abandoned) on the first visit were visited a second time later in the same day; if the occupants could not be found on the second visit or if they refused to participate, the household was skipped and replaced with another household.

A standardized questionnaire was used to collect the following information for each individual: name, gender, age, household size and residence (prefecture and village/neighbourhood. Malaria-related indicators included: reported history of fever/malaria, LLIN availability and use. Additional clinical data included: axillary temperature, thick blood smears and RDT malaria diagnosis. The same questionnaire was used for all survey rounds.

All interviews were conducted in French or the local language according to the preference of the participants, and data collection was supervised and monitored daily by one of two team leaders.

### Data entry and analysis

Data were double entered using EpiData (version 3.1, Odense, Denmark) and statistical analysis was performed using Stata (version 12.1, College Station, Texas). A symptomatic malaria infection was defined as a survey participant with a positive malaria rapid diagnostic test and an axillary temperature ≥37.5 °C on the day of the survey and/or a self-reported history of fever in the 24 h prior to the survey. Intervention coverage was estimated as: (1) the proportion of survey participants reporting an episode of fever in the month prior to the survey and seeking treatment from a health facility or CHW, and (2) the proportion of survey participants reporting LLIN ownership.

Malaria parasite prevalence (*Plasmodium falciparum*) according to RDT was calculated as the proportion of participants with positive RDT results among all participants tested. The proportion of participants fulfilling the definition of symptomatic (above) was calculated among all participants tested. Both variables were calculated separately for each survey round, sous-préfecture and by age group (1–59 months, 5–14, ≥15 years) using the Horvitz–Thompson estimator. Variance of the estimates was computed using the linearization method [14]. Proportions of participants seeking treatment from a health facility or CHW, in addition to the proportion of participants that reported owning an LLIN were estimated by sous-préfecture and by survey (April 2011 and February 2013).

Chi squared test was used to test differences between proportions. A *t* test or non-parametric test was applied to continuous variables when appropriate. Logistic regressions models were used to analyze temporal trends in malaria parasite prevalence. All test results were corrected to account for clustering in the sample design.

### Ethical considerations

The study proposal was reviewed and approved by the Médecins Sans Frontières Ethics Review Board (1028) and the Ethical Review Board of Guinea (02/CNERS/11). Participation was voluntary and written consent was obtained from each respondent or their caregiver before conducting the survey. If the participant was not literate, they were asked to make a cross instead of a signature. All personal data was anonymized and kept confidential, no individual identifiers were entered in the final database. Participants who tested RDT positive on the day of the survey were treated with ACT according to the national malaria treatment protocol.

## Results

### Individual and household characteristics

In total, 35,123 individuals participated in all surveys, approximately 7024 per survey. The median number of residents per household was eight and survey participants were predominately female (Table 1). The level of education of the head of household (HH) was generally low, with the majority reporting no formal education. Most survey participants (91 %) lived in dwellings with mud brick walls and a tin roof. There were no significant differences in demographic indicators across sous-prefectures or survey rounds.

**Table 1:** Household and individual characteristics of study population by sous-prefecture and survey period

Household characteristics	2011				2012				2013	
Area	N	April	N	August	N	February	N	August	N	February
*Median household size, n (range)*										
Guéckédou City	1762	7 (1–22)	1908	8 (3–30)	1694	8 (3–20)	1729	7 (2–17)	1733	8 (1–17)
Tékoulo	1690	7 (2–30)	1829	8 (3–26)	1684	8 (3–25)	1690	7 (3–22)	1694	7 (2–15)
Guendembou	1798	8 (2–20)	1880	8 (2–19)	1661	7 (1–20)	1730	6 (1–30)	1763	8 (3–16)
Koundou	1697	8 (2–24)	2016	8 (3–29)	1687	8 (4–21)	1750	7 (2–25)	1730	8 (1–25)
*Education level* + *, n (%)*										
Guéckédou City	272	165 (61 %)	297	152 (51 %)	317	186 (59 %)	301	153 (51 %)	275	124 (45 %)
Tékoulo	262	149 (57 %)	285	196 (69 %)	318	215 (68 %)	285	181 (64 %)	281	154 (55 %)
Guendembou	287	169 (59 %)	325	177 (54 %)	323	230 (71 %)	293	135 (46 %)	271	147 (54 %)
Koundou	260	173 (67 %)	320	219 (68 %)	305	239 (78 %)	282	192 (68 %)	291	186 (64 %)
*House structure* ^*a*^, *% (95* *% CI)*										
Guéckédou City	272	239 (88 %)	297	298 (100 %)	317	265 (84 %)	301	289 (96 %)	275	250 (91 %)
Tékoulo	262	241 (92 %)	285	283 (99 %)	318	279 (88 %)	285	259 (91 %)	281	198 (70 %)
Guendembou	287	270 (94 %)	325	321 (99 %)	323	303 (94 %)	293	273 (93 %)	271	259 (96 %)
Koundou	260	224 (86 %)	320	319 (99 %)	305	250 (82 %)	282	253 (90 %)	291	234 (80 %)
*Median age: median, (interquartile range)*										
Guéckédou City										
Overall	1762	11 (21.5)	1908	10 (19.5)	1694	18.2 (20.6)	1729	18.6 (19.9)	1733	18.2 (20.2)
1–59 months	505	0.3 (0.3)	504	0.36 (0.2)	499	0.36 (0.3)	391	0.36 (0.3)	512	0.36 (0.3)
5–14 years	555	9 (5)	732	9 (4)	464	9 (6)	592	8 (5)	447	8 (5)
≥15 years	701	29 (24)	672	29 (24)	731	30 (30)	746	30 (28)	774	30 (28)
Tékoulo										
Overall	1690	10 (27.5)	1829	10 (29.5)	1684	18.6 (19.9)	1690	19.6 (20.5)	1694	18.5 (20.1)
1–59 months	495	0.36 (0.3)	556	0.35 (0.32)	512	0.36 (0.3)	403	0.36 (0.3)	475	0.36 (0.2)
5–14 years	495	8 (4)	513	8 (5)	420	8 (4)	525	8 (4)	481	7 (4)
≥15 years	700	34 (25)	760	35 (21.5)	752	35 (20)	762	35 (26)	737	34 (24)
Guendembou										
Overall	1798	9 (29.5)	1880	8 (24.5)	1661	19.3 (20.7)	1730	19.2 (20.6)	1763	18.2 (20.3)
1–59 months	628	0.36 (0.3)	633	0.36 (0.3)	562	0.36 (0.3)	464	0.28 (0.3)	542	0.36 (0.3)
5–14 years	432	8 (5)	549	7 (4)	314	7 (4)	493	8 (4)	459	7 (3)
≥15 years	738	34.5 (20)	698	32 (23)	785	36 (22)	773	35 (24)	761	35 (24)
Koundou										
Overall	1697	11 (29.5)	2016	17.2 (29.5)	1687	19.6 (20.8)	1750	19.2 (20.9)	1730	19.8 (20.7)
1–59 months	522	0.36 (0.3)	567	0.36 (0.3)	466	0.36 (0.3)	460	0.36 (0.2)	450	0.36 (0.3)
5–14 years	426	8 (5)	610	8 (5)	475	7 (4)	524	7 (4)	493	7 (4)
≥15 years	749	35 (23)	839	32 (21)	746	38 (24)	766	35 (27)	787	35 (24)
*Sex, n (% male)*										
Guéckédou City	1762	742 (42 %)	1908	800 (42 %)	1694	689 (41 %)	1729	739 (43 %)	1733	698 (40 %)
Tékoulo	1690	798 (47 %)	1829	890 (49 %)	1684	772 (46 %)	1690	774 (46 %)	1694	757 (45 %)
Guendembou	1798	741 (41 %)	1880	844 (45 %)	1661	707 (43 %)	1730	790 (46 %)	1763	749 (42 %)
Koundou	1697	767 (45 %)	2016	949 (47 %)	1687	800 (47 %)	1750	865 (50 %)	1730	737 (43 %)

+ Highest education attained by the head of household, % none

^a^Walls made of mud brick and roof made of iron sheet vs other

### Malaria parasite prevalence

**Fig. 3:**
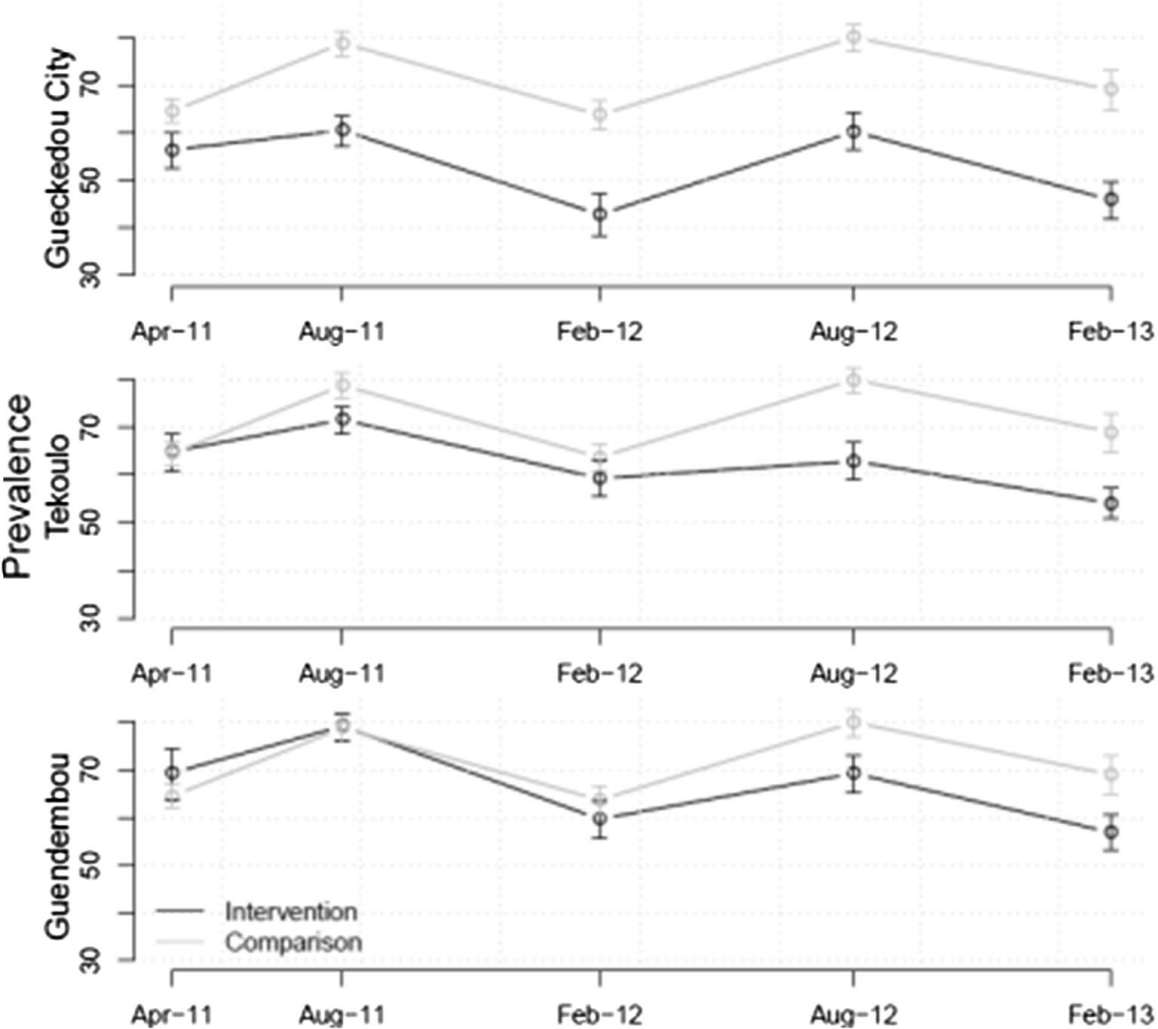
Malaria parasite prevalence according to rapid diagnostic test by Sous-prefecture and survey period

In April 2011, the prevalence of *P. falciparum* malaria infection according to RDT was over 55 % in all Sous-préfectures surveyed. In the first survey (April 2011), malaria parasite prevalence was significantly higher in the rural comparison sous-préfecture Koundou (64.5 %, 95 % CI 62–66.9) than in urban Guéckédou City (56.4 %, 95 % CI 52.6–60.1), p < 0.001. There was no significant difference between Tékoulo or Guendembou and the comparison Sous-préfecture (Fig. 3).

As seen in Fig. 3, after 2.5 years of the intervention, malaria parasite prevalence decreased significantly in all intervention sous-préfectures; Guéckédou City (45.9 %, 95 % CI 42.0–49.8, p < 0.01), Tékoulo (54.1 %, 95 % CI 50.9–57.3, p < 0.01) and Guendembou (56.9 %, 95 % CI 53.1–60.5, p < 0.01), while it increased insignificantly in the comparison sous-préfecture (69 %, 95 % CI 64.7–73.0, p = 0.06). The same trend was seen in the microscopy data (see Additional file 1).

Malaria parasite prevalence was consistently higher in children 5–14 years of age, followed by children 1–59 months of age and individuals ≥15 years of age in all sous-préfectures (Table 2). From April 2011 to February 2013, malaria parasite prevalence decreased significantly across all intervention sous-préfectures for children 1–59 months and 5–14 years. However, there was no significant change in malaria parasite prevalence for adults ≥15 years in any of the intervention sous-préfectures. No significant changes in malaria parasite prevalence by age group were seen in the comparison sous-préfecture during the same period (Table 2).

**Table 2:** Malaria parasite prevalence according to rapid diagnostic test by age group, sous-prefecture and survey period

	April 2011 % (95 % CI)	August 2011 % (95 % CI)	February 2012 % (95 % CI)	August 2012 % (95 % CI)	February 2013 % (95 % CI)
Guéckédou City					
1–59 months	54.8 (49.7–59.9)	60.7 (55.3–66.0)	40.0 (34.0–46.0)	54.4 (48.7–60.1)	44.3 (39.2–49.3)
5–14 years	78.3 (74.5–82.2)	75.9 (72.1–79.7)	65.9 (59.6–72.2)	80.2 (76.1–84.2)	67.3 (62.2–72.4)
≥15 years	40.3 (34.6–46.0)	43.6 (39.5–47.6)	29.9 (25.7–34.1)	47.4 (42.2–52.6)	34.6 (29.9–39.2)
Tékoulo					
1–59 months	67.9 (63.0–72.6)	75.3 (70.8–79.8)	63.0 (58.0–68.1)	61.7 (55.3–68.2)	54.3 (48.4–60.2)
5–14 years	89.4 (86.7–92.2)	92.0 (89.2–94.7)	84.5 (80.0–88.9)	88.3 (85.4–91.2)	78.3 (73.4–83.2)
≥15 years	45.4 (39.6–51.1)	55.1 (51.3–58.8)	42.6 (37.6–47.7)	46.0 (40.8–51.2)	38.3 (34.2–42.5)
Guendembou					
1–59 months	79.2 (74.8–83.7)	84.3 (81.3–87.4)	67.2 (61.8–72.7)	73.4 (67.2–79.6)	56.0 (49.8–62.2)
5–14 years	93.0 (90.4–95.6)	95.0 (92.8–97.3)	86.9 (82.8–91.0)	91.1 (88.2–93.9 %)	85.8 (82.0–89.6)
≥15 years	47.1 (39.5–54.8)	62.0 (57.3–66.7)	43.5 (39.0–48.0)	52.5 (48.1–58.8)	40.0 (36.1–44.0)
Koundou (comparison)					
1–59 months	73.9 (69.0–78.8)	85.1 (81.2–89.1)	68.2 (63.3–73.1)	88.0 (84.3–91.7)	78.0 (72.8–83.1)
5–14 years	89.4 (86.2–92.5)	93.7 (91.4–96.0)	91.1 (88.0–94.2)	95.2 (93.3–97.1)	90.4 (87.1–93.7)
≥15 years	43.7 (38.1–49.4)	63.7 (60.1–67.3)	43.5 (39.3–47.7)	64.7 (60.4–69.0)	50.4 (44.9–55.9)

### Symptomatic malaria infections

In April 2011 over 45 % of RDT positive participants had a symptomatic malaria infection: 46.3 % (95 % CI 38.1–54.4) in Koundou, 47.6 % (95 % CI 38.8–56.4) in Guéckédou City, 50.5 % (95 % CI 41.6–58.8) in Tékoulo and 49.6 % (95 % CI 42.4–56.8) in Guendembou. Compared to February 2013, there were no significant changes in the proportion of symptomatic malaria infectious by sous-préfecture or among children under 5 years of age. Overall, the proportion of symptomatic participants generally deceased with increasing age as seen in Table 3.

**Table 3:** Proportion of symptomatic, malaria rapid diagnostic test positive, participants by age, survey period and sous-prefecture

	April 2011 % (95 % CI)	August 2011 % (95 % CI)	February 2012 % (95 % CI)	August 2012 % (95 % CI)	February 2013 % (95 % CI)
Guéckédou City					
Overall	47.6 (38.8–56.4)	55.0 (43.5–66.5)	44.9 (35.7–54.1)	30.4 (18.8–41.9)	47.4 (35.1–59.8)
1–59 months	51.6 (41.5–61.6)	58.1 (47.2–69.1)	45.0 (34.0–55.9)	39.4 (26.9–51.9)	53.7 (40.9–66.5)
5–14 years	48.5 (37.8–59.1)	54.8 (42.4–67.2)	48.6 (37.8–59.5)	26.7 (15.2–38.2)	45.1 (31.5–58.7)
≥15 years	42.4 (33.3–51.4)	52.2 (38.0–66.3)	39.7 (30.4–49.0)	29.9 (16.4–43.4)	44.7 (30.5–59.0)
Tékoulo					
Overall	50.5 (42.6–58.8)	51.6 (42.5–60.7)	50.2 (41.6–58.8)	35.5 (21.9–49.0)	33.8 (24.2–43.5)
1–59 months	51.4 (42.2–60.7)	60.6 (52.0–69.2)	54.1 (44.5–63.7)	46.9 (33.0–60.8)	36.4 (26.8–46.0)
5–14 years	48.5 (39.4–57.5)	47.2 (35.9–58.5)	54.0 (43.4–64.7)	34.4 (19.7–49.1)	32.0 (20.4–43.6)
≥15 years	52.2 (42.8–61.5)	47.7 (37.8–57.6)	42.0 (31.6–52.4)	28.7 (15.7–41.8)	33.9 (22.7–45.1)
Guendembou					
Overall	49.6 (42.4–56.8)	52.7 (42.3–63.2)	45.7 (35.9–55.5)	31.6 (22.1–41.0)	41.5 (31.2–51.9)
1–59 months	51.6 (43.9–59.2)	55.8 (46.0–65.5)	41.1 (31.6–50.4)	41.9 (31.3–52.5)	42.7 (30.3–55.1)
5–14 years	49.7 (40.8–58.6)	51.7 (39.0–64.3)	52.7 (41.8–63.6)	29.3 (18.6–39.9)	39.8 (28.1–51.5)
≥15 years	46.8 (37.5–56.0)	50.3 (37.4–63.1)	45.3 (32.2–58.3)	25.7 (14.6–36.8)	42.6 (30.4–54.8)
Koundou (comparison)					
Overall	46.3 (38.1–54.4)	47.2 (38.2–56.1)	53.6 (42.9–64.3)	44.0 (34.6–53.5)	45.3 (34.6–53.5)
1–59 months	48.4 (38.4–58.4)	56.3 (48.2–64.4)	52.5 (41.3–63.6)	53.3 (44.0–62.5)	48.7 (37.4–59.9)
5–14 years	48.2 (39.3–57.2)	45.4 (34.5–56.3)	57.9 (45.4–70.4)	40.2 (29.2–51.3)	42.6 (29.7–55.4)
≥15 years	41.4 (30.8–52.0)	40.9 (30.9–50.9)	48.9 (37.7–60.0)	40.3 (30.1–50.5)	45.3 (32.5–58.1)

### Fevers and treatment-seeking behaviour

**Fig. 4:**
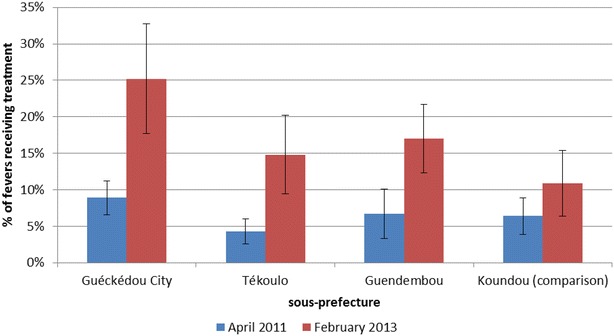
Treatment-seeking behaviour by sous-prefecture and survey period

As seen in Fig. 4, the proportion of participants who reported a history of fever in the month prior to the interview and who sought treatment at a health facility or from a CHW was low in April 2011: 6.4 % (95 % CI 3.9–8.9) in Koundou, 8.9 % (95 % CI 6.6–11.1) in Guéckédou City, 4.3 % (95 % CI 2.6–5.9) in Tékoulo and 6.7 % (95 % CI 3.9–8.9) in Guendembou. By February 2013, there was a significant increase in treatment seeking in the intervention sous-préfectures, Guéckédou City (25.2 %, 95 % CI 17.7–32.6, p < 0.001) Tékoulo (14.8 %, 95 % CI 9.4–20.2, p < 0.001) and Guendembou (17.0 %, 95 % CI 12.3–21.7, p = 0.001), while there was no significant change in the comparison sous-préfecture (10.9 %, 95 % CI 6.4–15.5, p = 0.086).

### LLIN ownership and use

**Fig. 5:**
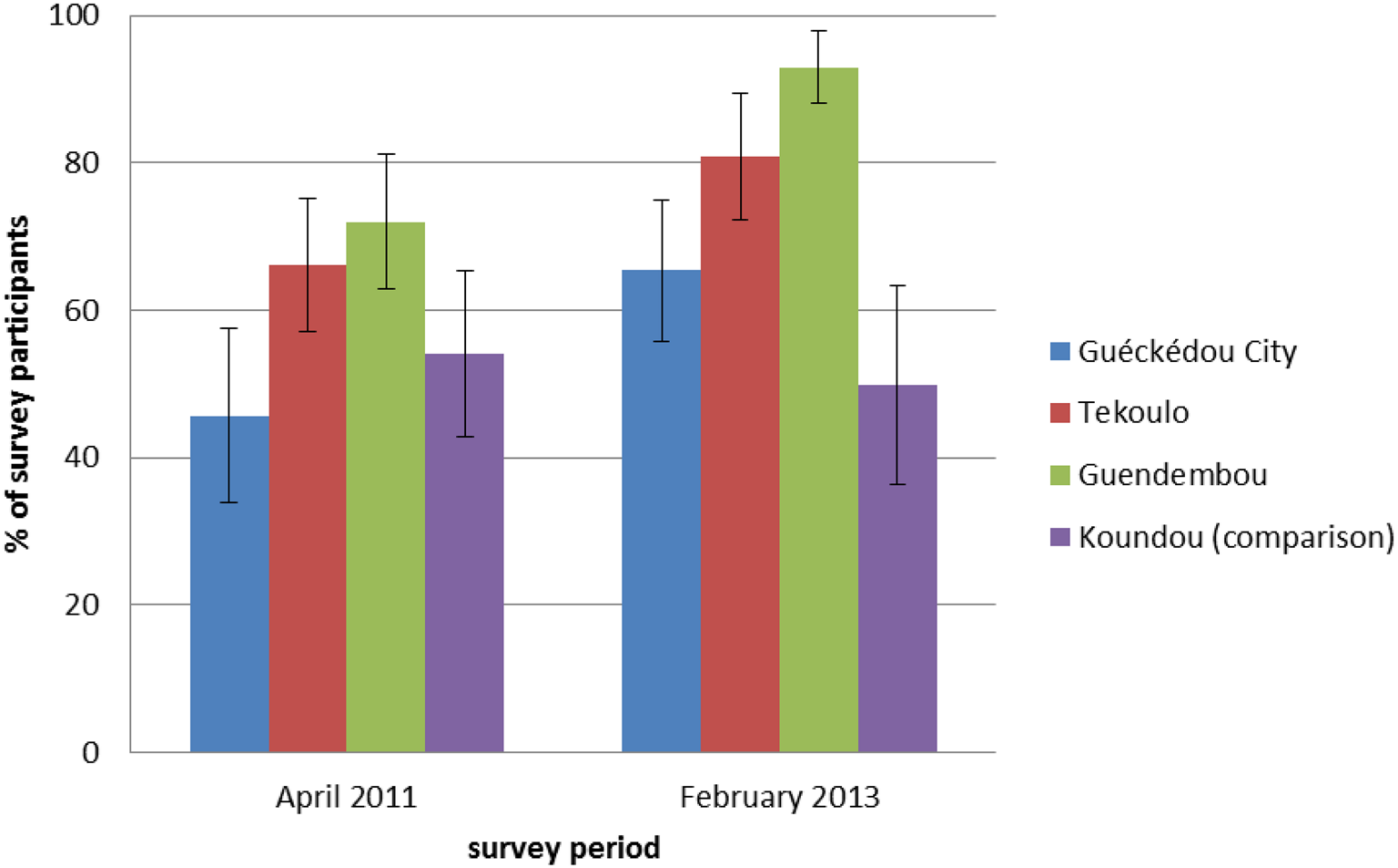
Reported LLIN ownership by Sous-prefecture and survey period

Over 40 % of survey participants reported owning a LLIN in April 2011. Reported LLIN ownership was higher in the rural sous-préfectures than in the urban sous-préfecture (Fig. 5). After the mass LLIN distribution was carried out in the intervention sous-préfectures in April 2012 the proportion of respondents who reported owning an LLIN in February 2013 increased significantly in all intervention sous-préfectures (45.7 to 65.4 % in Guéckédou City, p = 0.011; 66.2 to 80.8 % in Tékoulo, p = 0.022; 72.0 to 92.9 % in Guendembou, p < 0.001). No significant change in reported LLIN ownership was observed in Koundou (54.1 to 49.8 %, p = 0.631). Among participants who reported owning an LLIN in April 2011, reported LLIN use was over 94 % in all areas (range 94.5–99.4 %) and increased to over 98 % (range 98.0–99.4 %) in all sous-préfectures in February 2013.

## Discussion

Data from repeated cross-sectional surveys was used to assess the impact and coverage of a multi-component malaria control intervention in a hyperendemic region of Guinea. These results document improved coverage and a reduction in *P. falciparum* malaria parasite prevalence in the intervention sous-préfectures during the study period, April 2011 to February 2013. This contrasts with the comparison sous-préfecture where an increase in *P. falciparum* malaria parasite prevalence was observed during the same period.

Outside of trial conditions making direct comparisons between individual intervention sous-préfectures and the comparison sous-préfecture is difficult because malaria transmission dynamics likely vary across sous-préfectures and are not easily controlled. Indeed results from the first survey in April 2011 showed that malaria parasite prevalence was significantly different between urban Guéckédou City and the rural comparison sous-prefecture. However, despite not being directly comparable, the difference in the direction of the trends, an increase in malaria parasite prevalence in the comparison area and a decrease in Guéckédou City, remains indicative of the impact of the intervention package in the latter. Studies investigating the impact of multiple malaria control interventions have been carried out in other comparable settings, however implementation was frequently over smaller geographic areas, without a comparison group or used health facility-based surveillance data rather than malaria-specific surveys for evaluation [15–18].

Despite these encouraging findings, malaria parasite prevalence remains high in this region and is considerably higher in children (≤14 years of age) than adults. While none of the interventions specifically targeted children, malaria parasite prevalence in both groups of children (1–59 months and 5–14 years of age) decreased significantly over the study period in all intervention sous-préfectures albeit with seasonal fluctuations. Yet, there was no significant change in malaria parasite prevalence in individuals ≥15 years likely due to the presence of acquired protective immunity [19]. In the comparison sous-préfecture neither the overall change in malaria parasite prevalence nor age group specific changes were significant suggesting that decreases in malaria parasite prevalence in the intervention sous-préfectures were related to the intervention package. Nevertheless after 2.5 years of implementation malaria parasite prevalence remained over 44 % in children 0–59 months and 67 % in children 5–14 years of age. While this suggests that health interventions, including those for malaria, that focus on children under-five are justified in targeting this particularly vulnerable group it also demonstrates that interventions that focus on under-fives alone will be insufficient to control malaria transmission and that consideration should be given to enlarging the target age range [20, 21].

Estimates of malaria parasite prevalence varied depending on whether RDT or microscopy was used for evaluation. Although acknowledged that RDTs are less specific than microscopy for evaluation of malaria parasite prevalence [22, 23], the difference in the RDT and microscopy may also have resulted from the experience of the laboratory technicians and the condition of the slides when they were read. Notwithstanding, when repetitive testing and treatment of participants takes place, use of HRP2 tests should be discouraged due to their time to become negative [23], as their use may result in the unnecessary treatment of malaria negative participants.

The impact that treating at least 22,387 individuals over 2.5 years may have had on malaria parasite prevalence cannot be discounted. While treating so many people may have contributed to the decrease in malaria parasite prevalence documented here, surveys were carried out in newly selected clusters every 6 months. In this hyperendemic area it is unlikely that this focal, punctual administration of malaria treatment had a sustained impact on malaria parasite prevalence. In the absence of continued mass treatment it is unlikely that local transmission would not have been re-established in the 6 months between surveys.

Unlike previously reported decreases of symptomatic malaria infections after malaria control intervention implementation [15], there was no significant change in the prevalence of symptomatic infections in the intervention Sous-préfectures. Although the definition of symptomatic episodes used here is commonly used, this indicator may have been impacted in part by the self-reported nature of ‘history of fever’. Previous studies have shown that a caregivers determination of a febrile episode is frequently inaccurate [24, 25] making self-reported data less reliable than biomarkers such as measured temperature the day before the survey, gathered directly from participants. Furthermore, experiencing a febrile episode does not systematically indicate malaria infection as other common morbidities in the region also present with fever [26, 27]. Nevertheless despite its limitations, using fever or a history of fever as an indicator of uncomplicated malaria in areas of high endemicity is not without precedent [28–30].

Community health workers were recruited and trained to improve timely testing and treatment of cases of uncomplicated malaria and to refer cases of complicated malaria to the nearest health facility after administration of pre-referral treatment. Reported treatment seeking from a health facility or CHW, an indicator of intervention coverage, increased over time in all intervention sous-préfectures but was greatest in Guéckédou City, likely reflecting better access to health facilities in urban areas [31–33]. The increase in treatment-seeking from a health facility or CHW supports results from previous studies demonstrating that the availability of medication influences treatment-seeking behaviour for febrile illness [31, 34]. Despite the increase in treatment-seeking behaviour during the study period, it remains low, particularly in the rural sous-prefectures and suggests that additional activities targeting behaviour change may be needed to achieve and sustain greater impact.

In April 2011 none of the study sous-préfectures reported LLIN ownership of 80 % (2010 RBM target) [35]. After the April 2012 mass distribution, LLIN ownership had reached 80 % only in the two rural intervention sous-préfectures when measured in February 2013 not unlike the heterogeneity in coverage levels reported after a LLIN distribution in Ethiopia [36]. Low rates of reported LLIN ownership could be due to the manner in which the LLINs were distributed, at fixed points requiring individuals to leave their village in order to receive them. It may also be due to repurposing of LLINs. Indeed observations by the authors and anecdotal reports from survey teams described nets distributed in April 2012 for sale in markets and used as garden fencing and fishing nets. As the LLINS that were distributed by MSF were distinctive in size and color it is unlikely that there was confusion between these LLINS and other nets. However these experiences are not unique to this distribution [37]. Finally, the quality of the LLINs distributed could partially explain the elevated prevalence of malaria parasitaemia that persisted after 2.5 years of malaria control interventions. However, unlike reports of substandard LLINs distributed in Rwanda [38], the LLINs distributed in Guéckédou were procured by MSF and used by both the community and in MSF households and supported facilities. The authors are not aware of any reports of problems with the quality of the LLINs distributed. Nevertheless, in contrast to the relatively low LLIN coverage, reported use of LLINS was consistently high both prior to and after the LLIN distributions. For logistical reasons, implementation of the different components of the intervention package was heterogeneous and resulted in a key intervention, the mass distribution of LLINs, occurring over 12 months after implementation had begun. Admittedly a larger impact of the intervention package on malaria parasite prevalence may have been seen had LLINS been distributed at the beginning of the intervention.

The intervention package was constantly reviewed as it would be in ‘real life’ resulting in modifications of some activities. Consequently these results describe the impact of a multi-component malaria intervention implemented over a large geographic area and outside of trial conditions where extraneous factors are more easily controlled. It was not designed to quantify the impact of individual components of the intervention. Any indicator specific survey to survey variation is likely due to uncontrollable differences in implementation, the area of intervention and intervention components. While this variation presents a challenge for analysing programme impact, this approach also reflects the obstacles that malaria control programmes encounter with unavailable materials, and other problems, and highlights the difficulties of implementation on a large-scale outside of trial conditions [39].

This study has a number of limitations, particularly related to the realities of field implementation. First, information on all activities related to the intervention package in the study sous-préfectures was not systematically collected and intervention implementation was heterogeneous over time. Additionally, indicators that are known to impact malaria transmission, rainfall, village altitude, proximity to water, forest density and condition of LLINs, among others were not collected. Although it is difficult to know or record all changes in the intervention or these indicators in any field setting, similar studies in the future could attempt to more systematically collect this data. Second, the cross-sectional design of this study did not permit accounting for short-term fluctuations in parasitaemia however this effect is likely to have been small and not to have substantially impacted the overall results. Additionally estimates of malaria parasite prevalence varied according to which measure, RDT or microscopy was used for evaluation. The difference between the two measures could be due to the limitations of RDT performance [22] in addition to the prolonged time to become negative for HRP2 tests [23]. Third, because of the selection of new clusters for each survey round it was not possible to analyse spatial variations in malaria or determine particular parasite foci. Future studies could consider collecting data from the same geographic areas in order to account for spatial distribution. Finally, one indicator of intervention coverage was measured using an indicator heavily reliant on self-reported episodes of fever that encompasses more than malaria morbidity. Additionally, households were not purposefully visited in order to verify the information provided by the respondent concerning reported LLIN ownership. However, it is assumed that during the period of intervention other causes of common morbidities remain unchanged; reports of fever should provide a reliable indicator of malaria morbidity. As there were no major interventions aside from this intervention during the study period that could have led to the prevention of new fever cases this is a reasonable assumption.

## Conclusions

This study, which took place from 2011 to 2013 in Guéckédou, Guinea, shows an encouraging decrease in malaria parasite prevalence and an increase in treatment-seeking and ownership of LLINs. These results provide support for the RBM recommended malaria control interventions and add to a growing body of evidence that suggests that multi-component malaria interventions have a greater impact than singular interventions alone, at least in the short-term. While this malaria control intervention was ambitious and challenging to implement in a large, hyperendemic area with difficult access, these results show that wide-spread coverage can be obtained and intervention programmes can be monitored and adapted over time.

Although malaria parasite prevalence decreased, it remained unacceptably high even after 2.5 years of intervention, particularly in children ≤14 years of age. These results demonstrate the importance of reinforcing malaria control activities in hyperendemic areas, such as Guéckédou, in the long-term. They also highlight the need for development and implementation of age specific interventions and vector control measures to further reduce transmission.
